# Testing the McSad depression specific classification system in patients with somatic conditions: validity and performance

**DOI:** 10.1186/1477-7525-11-125

**Published:** 2013-07-26

**Authors:** Katerina Papageorgiou, Karin M Vermeulen, Maya J Schroevers, Erik Buskens, Adelita V Ranchor

**Affiliations:** 1 Health Psychology Section, University of Groningen, University Medical Center Groningen, Groningen, The Netherlands; 2 Department of Epidemiology, University of Groningen, University Medical Center Groningen, Groningen, The Netherlands

## Abstract

**Background:**

Valuations of depression are useful to evaluate depression interventions offered to patients with chronic somatic conditions. The only classification system to describe depression developed specifically for valuation purposes is the McSad, but it has not been used among somatic patients. The aim of this study was to test the construct validity of the McSad among diabetes and cancer patients and then to compare the McSad to the commonly used EuroQol – 5 Dimensions (EQ-5D^TM^) classification system. The comparison was expected to shed light on their capacity to reflect the range of depression states experienced by somatic patients.

**Methods:**

Cross-sectional data were collected online from 114 diabetes and 195 cancer patients; additionally, 241 cancer patients completed part of the survey on paper. Correlational analyses were performed to test the construct validity. Specifically, we hypothesized high correlations of the McSad domains with depression (Center for Epidemiological Studies Depression Scale (CES-D) and the Patient Health Questionnaire (PHQ-9)). We also expected low/moderate correlations with self-esteem (Rosenberg Self-Esteem scale - RSE) and extraversion (Eysenck Personality Questionnaire Extraversion scale - EPQ-e). Multiple linear regression analyses were run so that the proportion of variance in depression scores (CES-D, PHQ-9) explained by the McSad could be compared to the proportion explained by the EQ-5D classification system.

**Results:**

As expected, among all patients groups, we found moderate to high correlations for the McSad domains with the CES-D (.41 to .70) and the PHQ-9 (.52 to .76); we also found low to moderate correlations with the RSE (-.21 to .-48) and the EPQ-e (.18 to .31). Linear regression analyses showed that the McSad explained a greater proportion of variance in depression (CES-D, PHQ-9) (Diabetes: 73%, 82%; Cancer: 72%, 72%) than the EQ-5D classification system (Diabetes: 47%, 59%; Cancer: 51%, 47%).

**Conclusions:**

Findings support the construct validity of the McSad among patients with somatic conditions and demonstrate that it performs better than the EQ-5D classification system to reflect the range of depression states. For future valuation purposes, the McSad classification system could therefore be recommended to describe depression as experienced by patients with a chronic medical condition.

## Background

Depression is more prominent among patients with chronic medical conditions than in the general population [1,2]. Diabetes [3,4], cancer [5,6], heart disease [7], chronic obstructive pulmonary disease [8], and renal disease [9] are among the somatic conditions for which elevated rates of depression have been reported. Depressive symptoms represent a major burden for those patients, reducing their Health Related Quality of Life (HRQoL) and hampering their daily functioning [10-12]. It is important to assess depression specific HRQoL when evaluating the effectiveness of depression interventions offered to patients with somatic conditions.

Health state valuations (also called “utility values”, “utilities”, or “preferences”) are used to numerically express the HRQoL associated with a health state. Values represent “preferences” for specific health states and range from 0 (usually “worst imaginable health”) to 1 (usually “best imaginable health”). They provide a single metric that is applicable to different types of health conditions. These valuations are therefore convenient as HRQoL outcome measures for the economic evaluations of health care interventions [13]. Valuation occurs in two steps; first the health state is described/identified, and then it is valued. An appropriate and valid description of the state is a prerequisite for a valid valuation.

Generic utility measures such as the EQ-5D [14] and the SF-6D [15] are commonly used for the description and valuation of health states. The descriptive formats are often accompanied by scoring algorithms to assign indirect values to those states. As the classification systems are based on generic HRQoL domains, they are suitable for a wide range of somatic conditions. Although generic utility measures have also been used for valuations of depression [14,16-23], their suitability for mental health states has been questioned. The literature suggests a limited sensitivity of generic utility measures to capture changes in depression [24,25], but the evidence is neither consistent nor sufficient to draw definite conclusions [26,27].

The McSad is the only classification system developed specifically to generate descriptions of depression states for valuation purposes [28]. Similarly to generic systems such as the EQ-5D, the McSad is a brief self-report measure that puts a low burden on the respondent, thereby giving it high feasibility for large samples of respondents. The McSad consists of six depression domains defined on the basis of DSM III, each comprising four levels of functioning. It can therefore generate a large number of profiles for depression and reveal subtle differences among them. Its content validity was determined by reviewing experts’ evaluations. Its construct validity was demonstrated by consistent relationships between the severity of depression and the valuations provided by a population of outpatients with remission in their depression. The values for depression states generated by the McSad [28] have been used as outcome indices in a number of economic studies of depression interventions [29-34].

The McSad also seems suitable for patients with chronic medical conditions, though it has not been used before in this population. Its suitability thus needs to be demonstrated, starting with its classification system. It should be tested prior to and independent of its application for valuation purposes [35]. Specifically, the McSad may be deemed appropriate to describe depression among patients with somatic conditions for valuation purposes when it meets two conditions: first, it must have validity; second, it must perform better than commonly used generic measures.

This cross-sectional study examines the properties of the Dutch version of the McSad classification system to reflect depression among patients with chronic somatic conditions. Our first aim was to test the construct validity (convergent and divergent) by formulating and testing hypotheses regarding relationships of the McSad with selected measures. Our second aim was to test the capacity of the McSad to reflect the range of depression states, compared to the EQ-5D classification system (CS).

## Method

### Participants and data collection

The following inclusion criteria were used: aged 18 to 80 years; self-reported diagnosis of diabetes (Type I or II) or cancer (any type); and signed informed consent. For the sake of generalization, we included two separate etiology groups: diabetes and cancer. While both conditions carry a high risk for depressive symptomatology, they differ considerably. For example, cancer is directly related to the risk of death, and its treatment is often intensive, e.g. surgery or chemotherapy [36]. Diabetes, on the other hand, is not directly related to risk of death, and its primary treatment mostly involves management of glucose levels [37].

An online survey was carried out from September 2010 through November 2011 using the Unipark software package (http://www.unipark.com). Diabetes patients were recruited through the http://www.dvn.nl site (Diabetes Association Netherlands). Cancer patients were recruited through the following sites: http://www.nfk.nl (Dutch Federation of Cancer Patient Organizations); http://www.borstkankertrefpunt.hyves.nl (forum for people dealing with breast cancer issues); http://www.olijf.nl (network for women with gynecological cancer). The patient associations behind these sites were contacted to inform them about the study and request them to host the survey. Once permission was granted, the web page posted a link to the survey, stating the affiliation and the general purpose of the study. Anyone who visited the site could see the posting; those choosing to follow the link could read more about the purpose of the study and eligibility to participate in it. Those who signed the informed consent could go on to complete the survey.

For the sake of simplicity, we refer to the online participants as the Diabetes and the Cancer groups. An additional group of cancer patients completed a brief version of the survey on paper. We refer to the latter participants as the Cancer-paper group. The reason to have an additional group was to cross-check some of our findings in patients that differed in terms of administration (paper vs. computer) and recruitment method (physician instead of internet). Members of the Cancer-paper group were approached by their physicians at the Radiotherapy Department of the University Medical Center Groningen during the period April to November 2011. They were contacted in the context of an ongoing project of the Health Psychology Section, University Medical Center Groningen. Patients giving their informed consent were sent a paper survey by post.

Participants in the Diabetes and Cancer groups first provided information on their gender, age, years since diagnosis, and the existence of other important medical conditions. Next, the McSad classification system was presented as a self-report health check list. The respondents were asked to identify their level of functioning within the previous week. Finally, they completed depression questionnaires and provided information for other scales. Participants in the Cancer-paper group first answered the personal and disease-related questions and then provided the other information, though only on the McSad and a depression scale.

### McSad classification system

The McSad was designed to describe Major Unipolar Depression for valuation purposes. It distinguishes six domains of distinct depressive symptoms, in accordance with the DSM-III [38]. Each one (Emotion, Self-appraisal, Cognition, Physiology, Behavior, and Role function) recognizes four levels of dysfunctioning: no (1), mild (2), moderate (3), and severe (4). The Emotion domain combines symptoms of a blue mood and a loss of interest (example of mild dysfunctioning: “*Feel more down (or sad, blue, depressed) than usual and don’t enjoy things as usual*”). The Self-appraisal domain concerns how one views the self and the world (example of mild dysfunctioning: “*Don’t feel very good about myself these days and often see the down-side of everything*”). The Cognition domain describes cognitive performance such as concentration, memory, and decision-making (example of mild dysfunctioning: “*Have some trouble concentrating and remembering these days, and it seems harder to make decisions*”). The Physiology domain refers to physical symptoms of depression such as sleep, energy, and appetite (example of mild dysfunctioning: “*Sleep is quite troublesome these days. Don’t have quite the normal get up and go, and have less of an appetite*”). The Behavior domain relates to symptoms of psychomotor agitation/retardation and, in the more severe categories, to suicidal ideation (example of mild dysfunctioning: “*Things are more of a chore these days and at times I feel sluggish or agitated*”). Finally, the Role function domain addresses performance in work, home, or social settings (example of mild dysfunctioning: “*Able to function okay at work, home, school, or with friends but often don’t enjoy what I am doing, and/or feel more withdrawn lately*”). The full version of this classification system is available in the publication by Bennett et al. [28]. McSad is a self-report instrument. For each of the six domains, respondents are asked to choose the one level that best describes how they had functioned during the past week. The answers generate a single metric representing a descriptive profile (for example, profile 232322 describes mild dysfunctioning in Emotion, Cognition, Behavior, and Role function and moderate dysfunctioning in Self-appraisal and Physiology). All possible combinations of the four levels in the six domains would generate 4096 (4^6^) unique descriptive profiles of depression states.

For the purposes of the current study, the McSad was translated into Dutch, using established forward-backward translation guidelines [39,40], i.e., translated into Dutch and then back to English. The Dutch translation was made by a group of researchers at the Health Psychology Section of the University Medical Center Groningen (one professor, two senior researchers, and one research assistant), all of whom are native Dutch speakers and fluent in English, with extensive experience in using depression scales for research among patients with somatic conditions. Then an expert in English with no prior knowledge of the questionnaire was engaged for the back translation. The translated versions were discussed in order to reach consensus on slight differences in wording. Content validity was assessed by the same group; they assessed whether all depression symptoms are covered by the McSad and how consistently the four levels reflect the levels of dysfunctioning in each domain. Given that the McSad was developed on the basis of DSM criteria, we first made a point-by-point comparison of the DSM and the McSad. Furthermore, we compared the McSad with depression scales commonly used among patients with somatic conditions (PHQ-9, CES-D, and HADS). After assessing the content validity, minor changes were made in levels 3 and 4 of the Behavior domain with regard to suicidal tendency, as the way they had been formulated was considered extremely negative.

In order to test the McSad psychometrically, each domain was assigned a score from 1 (*no dysfunctioning*) to 4 (*severe dysfunctioning*).

### Construct validity

To test construct validity of the McSad, we examined the correlations of the McSad domains (i.e. domain scores) with depression scales (i.e. total scores), as well as with other scales measuring constructs related to depression, that is self-esteem and extraversion. In light of our conceptual framework, based on theory and previous research, we formulated working hypotheses regarding the construct validity of the McSad. Specifically, we hypothesized higher correlations of the McSad domains with the depression scales (convergent validity) than with self-esteem and extraversion scales (divergent validity).

### Convergent validity

#### Hypotheses

We hypothesized that the aggregate scores for the Cancer and Diabetes groups on all McSad domains would correlate strongly with their total scores on the two depression scales investigated here, i.e., the Center for Epidemiological Studies Depression Scale (CES-D) [41,42] and the Patient Health Questionnaire (PHQ-9) [43]. We also looked into the correlation between the McSad domains and the CES-D for the Cancer-paper group. As differences in the administration and recruitment method were not expected to exert any influence on the hypothesized relationships of the McSad with the depression scales, we expected to find strong correlations in this group too.

#### Measures

The Patient Health Questionnaire (PHQ-9) is a depression scale consisting of nine items. These correspond to DSM depression criteria such as blue mood and sleep problems. Items are presented as questions about the frequency of depression symptoms within the past two weeks. Answer categories range from 0 (“not at all”) to 3 (“almost every day”). The item scores are summed to calculate the total depression score, which ranges from 0 (no symptoms) to 27 (highest level of depression). The PHQ-9 has been demonstrated to be a reliable and valid instrument for screening for depression and for assessing its severity, also among populations with a background of medical issues. A mean score of 5.08 on the PHQ-9 questionnaire has been reported for primary care patients [44]. The previously validated Dutch version of the PHQ-9 was used here [45]. In the current samples, internal consistency was good, as indicated by high Chronbach’s alphas of .90 and .83 in the Diabetes and Cancer groups, respectively.

The Center for Epidemiological Studies Depression Scale (CES-D) is a validated self-report scale for assessing depressive symptoms. It consists of 20 items representing symptoms of depression. These concern a depressed mood, feelings of guilt and worthlessness, feelings of helplessness, psychomotor retardation, loss of appetite, and sleep disturbance. The scale addresses the frequency of such symptoms within the last week. Items are scored on a four-point scale ranging from 0 (*rarely or none of the time*, i.e., *<1 day*) to 3 (*most or all of the time*, i.e., 5-7 days). Four of the items are phrased in reverse order and recoded accordingly. Item scores are summed to calculate the scale score, which ranges from 0 to 60, with higher scores representing higher levels of depression. The CES-D has also been validated among patients with chronic medical conditions [46,47]. An average CES-D score of about 12 has been reported for cancer patients under treatment [48,49]. The validated Dutch version of the scale was used here [50]. In the current samples, internal consistency was good, as indicated by high Chronbach’s alphas of .93, .89, and .85 in the Diabetes and Cancer and Cancer-paper groups, respectively.

### Divergent validity

#### Hypotheses

In accordance with the literature, we hypothesized that all six McSad domains would show moderate correlations with self-esteem [51,52] and weak correlations with extraversion [53]. We then examined the correlations of the McSad domains with Rosenberg’s self-esteem scale [54] and the Eysenck Personality Questionnaire – Extraversion scale [55].

#### Measures

Rosenberg’s Self-Esteem scale (RSE) is used to assess one’s level of self-esteem. It includes ten items related to self-esteem as reflected by respondents. For example, one item states, “I feel that I have a number of good qualities”. Items are answered on a four-point scale, ranging from 1 (“*strongly agree*”) to 4 (“*strongly disagree*”). Five items (1, 2, 4, 6, 7) are worded positively, the other five (3, 5, 8, 9, 10) negatively. Negative items are reverse-coded. A total scale score is calculated by summing the item scores, thus ranging from 10 to 40, with higher scores indicating higher self-esteem. The reliability and validity of this scale have been demonstrated [56].

The Eysenck Personality Questionnaire – Extraversion scale (EPQ-e) consists of 12 items to assess extraversion. These are posed as questions; for example, “Are you a talkative person?” The participants are asked to answer on a dichotomous scale (*yes*: 1; *no*: 0). The total score for the scale is calculated by summing the item scores; thus, the total can range from 0 to 12, with higher scores indicating higher extraversion. The psychometric properties of the EPQ have been established [57-59].

### Comparison of the McSad to the EQ-5D classification system

#### Hypothesis

To assess the capacity of the McSad to reflect the range of depression states experienced by patients with somatic conditions, we examined the degree of variability in depression scores that can be explained by the McSad and compared that outcome to the degree of variability in depression scores that can be explained by the EQ-5D CS [14]. We hypothesized that altogether the six McSad domains would account for a large proportion of variability in the depression scores assessed by the CES-D and the PHQ-9. Further, we expected this proportion to be larger than that explained by all the EQ-5D domains.

#### Measures

The EuroQol – 5 Dimensions (EQ-5D^TM^) is a generic health status measure. It consists of a classification system and a Visual Analogue Scale. The present study used only its classification system, which comprises five dimensions: mobility, self-care, daily activities, pain/discomfort, and anxiety/depression. Respondents are asked to choose the one level out of three (*no*, *moderate*, *severe problems*) that reflects their current functioning. Their scores on each domain are combined to generate descriptive profiles appropriate for valuation. In order to conduct psychometric testing of the EQ-5D CS, we used domain scores ranging from 1 (*no problems*) to 3 (*severe problems*).

### Statistical analysis

All the variables were examined for outliers, missing data, and normality. The Diabetes and Cancer groups were compared with respect to background variables by means of *t* tests and chi-square tests. Similarly, the Cancer-paper group was compared to the Cancer group.

The McSad was inspected to discern the distribution of levels and the number of different profiles identified among the Diabetes and Cancer groups.

Relationships of the McSad domains with the selected scales were assessed by means of a Spearman’s Rho correlation coefficient, as the assumption of normality of distribution was violated. Correlations higher than .50 were considered strong, those less than .30 weak [60].

A number of multiple linear regression analyses were performed to compare the McSad to the EQ-5D CS concerning their capacity to reflect the range of depression scores. The first regression analysis examined all six McSad domains, treating them as predictor variables. The answer categories were coded as dummy variables and the Enter method was used, while the total score on the CES-D was used as an outcome variable. The second regression analysis was similar to the first except that the PHQ-9 total score was used as an outcome. The third and fourth regression analyses were almost equivalent to the first two, though not quite; instead of using the McSad domains as predictor variables, the EQ-5D domains were used for that purpose.

All analyses were carried out separately for the Diabetes and Cancer groups. Data from the Cancer-paper group on the McSad and the CES-D were used only to cross-check convergent validity findings. Statistical significance was assumed for *p* < .05. Data were analyzed using SPSS, version 16 (SPSS Inc., Chicago IL).

## Results

### Participants & data description

**Figure 1:**
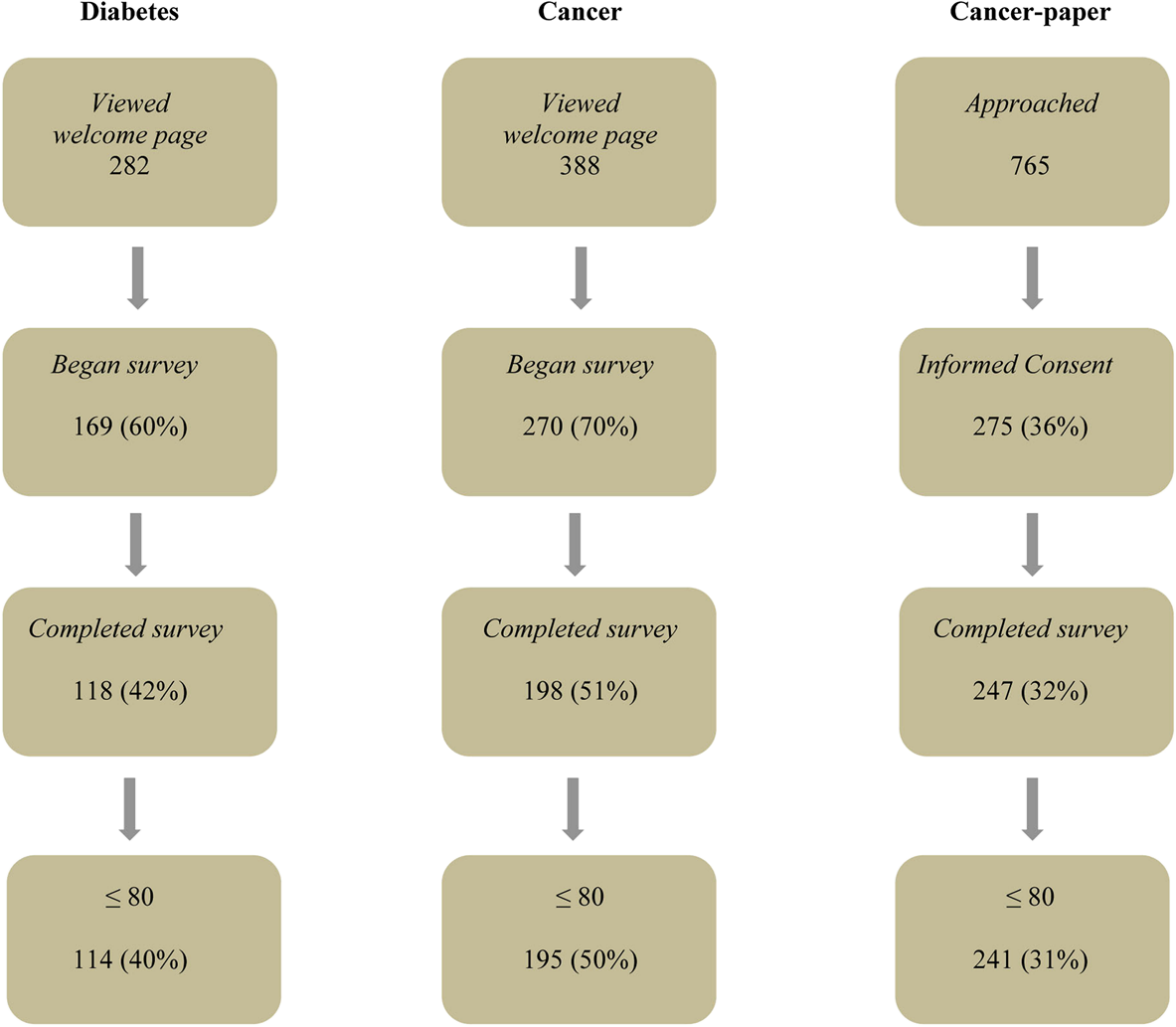
Study profile.

The sample for the survey consisted of 114 participants from the Diabetes and 195 from the Cancer group, as well as 241 from the Cancer-paper group. The profile of the study with respect to response and completion rates is presented in Figure 1. The characteristics of the participants, concerning personal and disease-related information as well as levels of depression, are displayed in Table 1. Women were in the majority in the Diabetes and Cancer groups (72% and 84% respectively), whereas men were in the majority (76%) in the Cancer-paper group. The mean age in all three groups was over 40, and the number of years since diagnosis varied considerably. Average levels of depression were found to be lower than the threshold for clinically relevant symptoms, with the exception of the Cancer group, but then only when they were assessed using the CES-D. The Cancer and Diabetes groups were found to be similar in terms of comorbidity rates and depression levels. However, participants in the Diabetes group were more often men, younger, and had been diagnosed longer ago than participants in the Cancer group. When compared to the Cancer group, participants in the Cancer-paper group were more often men, older, and experienced lower levels of depression.

**Table 1:** Characteristics of participants

	**Diabetes**	**Cancer**	***Diabetes vs. vancer***	**Cancer-paper**	**Cancer vs. cancer-paper**
N	114	195		241	
Age, years					
Mean (SD)	44 (14.1)	52 (10.9)	t (192) = 5.18**	65.3 (11.6)	t (412) = 11.7**
Range	20 - 73	22 - 77	*p < .000*	36-79	p *<* .000
Gender					
women	82 (72%)	163 (84%)	*χ*^*2*^*(1) = 6.36*	55 (24.2%)	χ^2^ (1) = 145**
			*p = .013*		p *<* .000
Years since diagnosis					
Median (IR)	13.5 (17)	3 (3)	t (128) = 8.52**	3 (3)	*t (412) = 2.0*
Range	0-56	1-31	*p < .000*	0-25	*p = .057*
Main co-morbidity					
COPD, asthma	9 (7.9%)	19 (9.7%)	*χ*^*2*^*(1) = .32*	17 (7%)	χ^2^ (1) = 1.08
			*p = .57*		p = .30
Heart	8 (7.0%)	6 (3%)	*χ*^*2*^*(1) = 2.52*	30 (12.3%)	χ^2^ (1) = 12.32**
			*p = .11*		p = .00
Rheumatoid arthritis	5 (4.4%)	14 (7.2%)	*χ*^*2*^*(1) = .46*	15 (6.2%)	χ^2^ (1) = 177
			*p = .50*		p = .70
CES-D (depression)					
Mean (SD)	14.31 (11.6)	16.09 (9.6)	*t (190,535) = 1.34*	9.9 (7.7)	t (352) = 7.0**
Range	0 – 50	0 – 45	*p = .181*	0-37	*p = .000*
PHQ-9 (depression)					
Mean (SD)	6.41 (6.03)	6.43 (4.69)	*t (187,889) = .071*	-	
Range	0 – 25	0 – 27	*p = .943*		

(*Columns in italics present results of t and chi-square tests concerning differences among groups).*

* p < .05 / ** p < .01.

Items were missing on the McSad for less than 3% of the cases for all groups, and listwise exclusion was used. No extreme outliers were identified for the included variables. For most of the variables, the distribution violated normality, so non-parametric tests were used.

**Figure 2:**
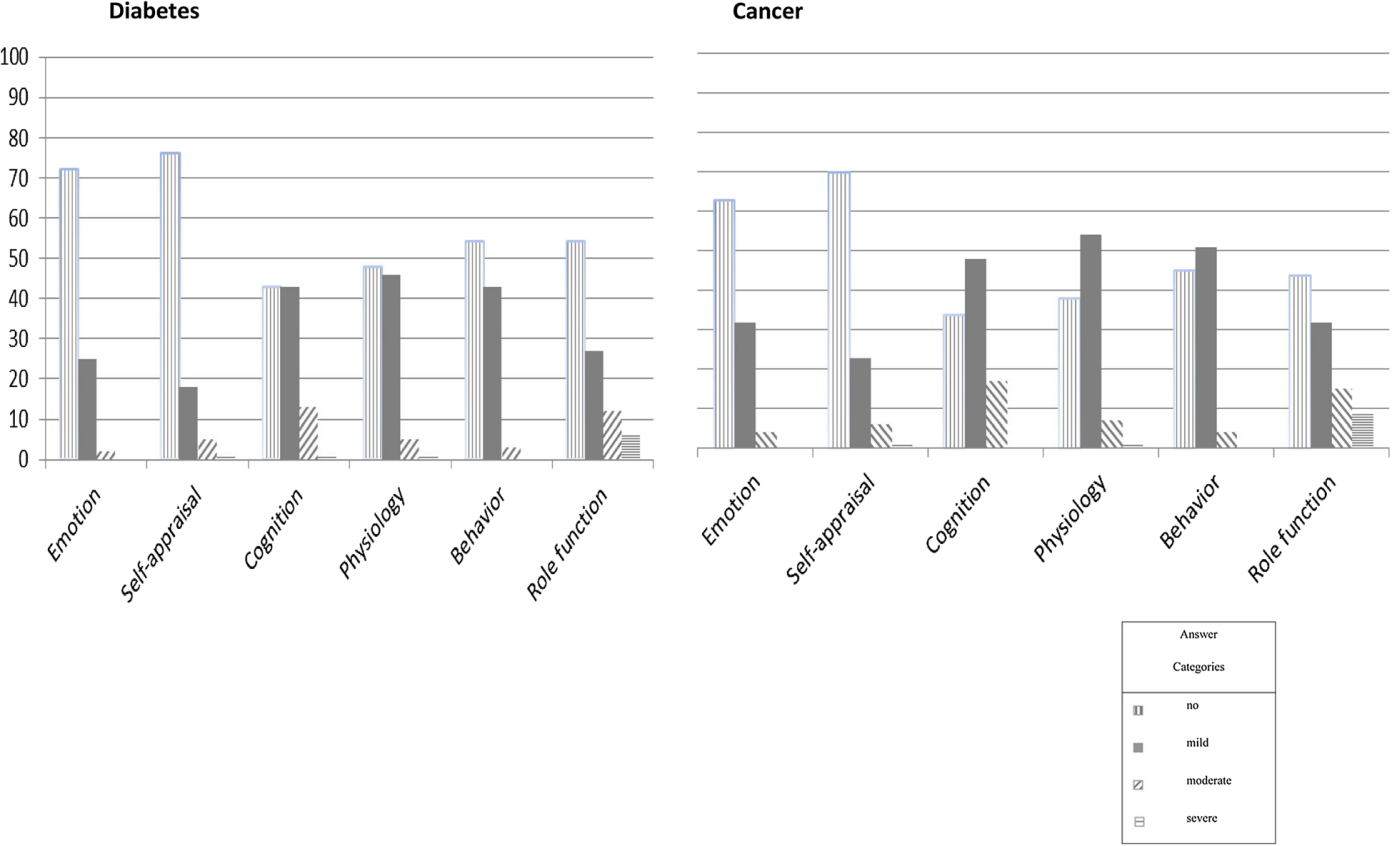
Distribution of answer categories (%) on the McSad domains.

The distribution of levels of severity on the McSad domains for the Diabetes and Cancer groups is displayed in Figure 2. It clearly shows an overall floor effect in levels for all six McSad domains and a variation from normality. Specifically, the percentage of answers falling into level 3 or 4 ranges from 2% in the Emotion domain to 18% in the Role function domain for the Diabetes group. For the Cancer group, answers in these higher levels range from 4% in the Behavior domain to 24% for Role function. Of the 114 participants in the Diabetes group, 77 reported at least mild dysfunctioning on at least one of the McSad domains. For these 77 respondents, a total of 53 different depression states were identified by the McSad. Similarly, 163 out of the 195 participants in the Cancer group reported at least mild dysfunctioning in at least one McSad domain; for these 163 patients, a total of 71 different depression states were detected.

### Construct validity

Table 2 summarizes the results on convergent and divergent correlations of the McSad domains with depression, self-esteem, and extraversion scales.

**Table 2:** Construct validity findings

	** Diabetes**				** Cancer**				**Cancer-paper**
**McSad domains**	**CES-D**	**PHQ-9**	**RSE**	**EPQ-e**	**CES-D**	**PHQ-9**	**RSE**	**EPQ-e**	**CES-D**
Emotion	.70**	.73**	-.43**	.24**	.64**	.52**	-.21**	.23**	.44**
Self-appraisal	.68**	.73**	-.48**	.26**	.61**	.52**	-.25**	.30**	.45**
Cognition	.67**	.72**	-.45**	.21**	.52**	.63**	-.25**	.20**	.43**
Physiology	.50**	.65**	-.32**	.16	.41**	.62**	-.10	.23**	.50**
Behavior	.68**	.76**	-.44**	.22**	.64**	.61**	-.29**	.22**	.52**
Role function	.68**	.74**	-.41**	.31**	.61**	.57**	-.28**	.18*	.47**
*Mean*	.65	.72	-.42	.23	.57	.58	-.23	.23	.47

Correlations (Spearman’s Rho) of McSad domains with depression (CES-D, PHQ-9), self-esteem (RSE) and extraversion (EPQ-e) scales.

* p < .05/** p < .01.

### Convergent validity

All the correlations between the McSad domains and depression scales were found to be significant. Correlations were strong for the Diabetes group (CES-D: .50 to .70; PHQ-9: .65 to .76) and moderate to strong for the Cancer group (CES-D: .41 to .64; PHQ-9: .52 to .63). Correlations between the McSad domains and the CES-D were found to be moderate to strong among the Cancer-paper group (.43 to .52).

#### Divergent validity

The correlations between the McSad domains and self-esteem were found to be statistically significant for both groups, though moderate in the Diabetes group (-.32 to -.48) and weak in the Cancer group (-.21 to -.29). The one exception was a non-significant relationship in the Physiology domain for the Cancer group. Correlations with extraversion were found to be significant though weak for both the Diabetes (.21 to .31) and the Cancer group (.18 to .30), with the exception of a non-significant correlation in the Physiology domain for the Diabetes group.

On the whole, all six McSad domains had stronger correlations with depression scales than with self-esteem and extraversion.

### Comparison of the McSad to the EQ-5D classification system

The results of the regression analyses are presented at Additional file 1: Table S1. Due to the very limited number of participants whose answers fell into the fourth level on the McSad domains (0 to 18 cases), levels 3 and 4 were merged.

Overall, the results indicate that for both the Diabetes and the Cancer groups, a substantially larger proportion of variance in depression scores (CES-D, PHQ-9) could be explained when the six McSad domains were used as predictor variables (Diabetes: 73%, 82%; Cancer: 72%, 72%) than when the EQ-5D domains were used (Diabetes: 47%, 59%; Cancer: 51%, 47%).

## Discussion

The McSad depression specific classification system seems to appropriate for use among patients with somatic conditions. However, it has not yet been validated in this population. Nor has its performance been compared to that of the commonly used EQ-5D classification system. In this study, we examined the McSad among diabetes and cancer patients.

Our findings support the expected construct validity of the McSad. All six McSad domains correlate either strongly or moderately with depression scales. In contrast, the observed moderate/weak associations of McSad domains with self-esteem and the weak associations with extraversion are in line with our hypotheses. Since the McSad demonstrates the types of associations that would be expected for a depression measure in light of previous research, we have found evidence in support of its validity. Furthermore, compared to the EQ-5D classification system, the performance of the McSad to reflect the range of depression scores appears considerably better. Therefore, the McSad seems to be a valid and appropriate classification system for describing depression among patients with somatic conditions.

Having found that the McSad, in addition to being valid, also appeared to perform better than the EQ-5D classification system in reflecting the range of depression states, we can recommend applying this instrument in valuations of depression that can subsequently be used in cost-utility analyses of psychological interventions for patients with somatic conditions. Optimally, the McSad could be used in combination with a generic utility measure in order to detect depression-specific as well as general health-related changes.

When interpreting the findings of this study, some of its limitations should be considered. Regarding the sample, the restricted number of participants with more severe levels of depression and the consequent floor effect observed in the McSad constrain our ability to draw conclusions on the capabilities of the McSad for reflecting more severe levels of depression. Given that the reported levels of depression were in accordance with what was expected in this population, the small number of participants with severe depression could be addressed by increasing the sample size. Furthermore, the relationships between the McSad and the other scales were examined for each McSad domain. Specifically, we examined the relationships between domains (e.g., Cognition) with a whole construct (e.g., Depression), which could partially explain finding correlations that were somewhat different than expected. On the other hand, this approach is appropriate for testing a multi-attribute classification system that was not designed to compute a total score. Investigation of two additional issues could further support the conclusions of the current study. First, it can currently not be ruled out that symptoms of depression included in the Physiology domain might overlap with symptoms of the somatic condition and/or its treatment. For example, apart from being a depression symptom, fatigue is one of the most persistent side-effects of cancer treatment [61]. Also, sleeping problems, included in the Physiology domain of the McSad, are also frequently experienced by patients with diabetes [62]. Such replication has been previously recognized in established depression scales such as the PHQ-9, in which psychometric testing favored inclusion of such symptoms in the scales. Secondly, a clinical interview, accepted as the “gold standard” for assessing depression, might be preferable for testing the validity of the McSad. However, use of self-report instruments allows us to obtain more information on differences in the severity of depression.

One strength of the current study is that the McSad was tested separately in groups of people with different somatic conditions, namely diabetes and cancer. Another is that it cross-validates some of those questions in an additional group of patients representing a different recruitment and administration method. Our conclusions are also strengthened by testing and confirming our hypotheses using two established depression scales (CES-D, PHQ-9).

Further studies on the McSad classification system among patients with somatic conditions could focus on its responsiveness, especially before and after an intervention that is known to meaningfully reduce depression. Comparison of the McSad to the EQ-5D classification system on their relative capacity to reflect changes is also warranted. Furthermore, the properties of the McSad classification system could be studied in different populations of patients with chronic somatic conditions associated with increased risk of depression, for example heart or COPD patients. Finally, the development of a scoring formula which can be used to attach values to all the states generated by the McSad classification system would make the instrument directly available for use in intervention evaluations. This could, in turn, make health valuation more applicable as an outcome measure in the mental health field, which is now lagging behind [63].

## Conclusions

We examined the McSad depression specific classification system among patients with somatic conditions. It proved to be valid and it appears to perform considerably better than the commonly used EQ-5D classification system. Given these findings, we conclude that the McSad is the instrument of choice to reflect depression among patients with somatic conditions for future valuation studies.

## Competing interests

The authors declare that they have no competing interests.

## Authors’ contributions

KP performed the data collection and statistical analyses and drafted the manuscript. All authors contributed to the conception and the design of the study and to writing the manuscript. All authors have read and approved the final manuscript.

## Supplementary Material

Additional file 1: Table S1 Results of Linear Regression analyses, with the McSad/EQ-5D answer categories (dummy variables) as predictors and the CES-D/PHQ-9 total scores as outcome variables. Click here for file
