# Supplementary material for: Testing the McSad depression specific classification system in patients with somatic conditions: validity and performance
Source: Health Qual Life Outcomes. 2013 Jul 26;11:125. doi: 10.1186/1477-7525-11-125 (PMC3735482; doi:10.1186/1477-7525-11-125)
Supplement: Additional file 1: Table S1 — Results of Linear Regression analyses, with the McSad/EQ-5D answer categories (dummy variables) as predictors and the CES-D/PHQ-9 total scores as outcome variables. [file 1477-7525-11-125-S1.docx]

**Table S1.** Results of Linear Regression analyses

|  | **Predictor** | **Outcome** | **R2** | **df** | **Anova F** |
| --- | --- | --- | --- | --- | --- |
|  | McSad | CES-D | .73 | 12, 94 | 21.47** |
| **Diabetes** |  | PHQ-9 | .82 | 12, 96 | 36.37 ** |
|  | EQ-5D | CES-D | .47 | 8, 98 | 12.817** |
|  |  | PHQ-9 | .59 | 9, 99 | 15.981** |
|  | McSad | CES-D | .72 | 12, 174 | 32.61** |
| **Cancer** |  | PHQ-9 | .72 | 12, 171 | 36.1** |
|  | EQ-5D | CES-D | .51 | 9, 177 | 20.20** |
|  |  | PHQ-9 | .47 | 9, 174 | 17.28** |
| *McSad / EQ-5D levels (dummy variables) as predictors and CES-D / PHQ-9 total scores as outcome variables.* | | | | | |
| ** p < .01 | | | | | |
